# Impact of COVID-19 on blood donation and supply in Africa

**DOI:** 10.4102/ajlm.v10i1.1408

**Published:** 2021-10-25

**Authors:** Kenneth B. David, Knovicks Simfukwe, Mohamed B. Musa, Steven Munharo, Don E. Lucero-Prisno

**Affiliations:** 1 Faculty of Pharmaceutical Sciences, Kaduna State University, Kaduna, Nigeria; 2 Hull York Medical School, University of Hull, Hull, United Kingdom; 3 School of Veterinary Medicine, University of Zambia, Lusaka, Zambia; 4 Faculty of Pharmacy, Omdurman Islamic University, Khartoum, Sudan; 5 Training and Research Unit of Excellence, College of Medicine, University of Malawi, Blantyre, Malawi; 6 Department of Global Health and Development, London School of Hygiene and Tropical Medicine, London, United Kingdom

## What is the problem? Blood donation shortages

As the coronavirus disease 2019 (COVID-19) continues to spread in Africa, unprecedented disruptions at all levels of human endeavours including healthcare delivery systems have been recorded.^1^ One of the major areas of healthcare systems affected is blood supply – a commodity needed for the survival of many patients. Blood and blood products such as cryoprecipitate, plasma, immune globulins, platelets, etc., are required for the management of medical conditions including but not limited to trauma, renal impairment, cancer, sickle cell anaemia, haemorrhagic shock, and other medical conditions related to acute or chronic loss of blood.

Despite the COVID-19 pandemic, health problems and accidents occur including maternal morbidities, malnutrition, blood-transmitted infectious diseases (HIV and AIDS, hepatitis C virus infection, hepatitis B virus infection, syphilis and anaemia-inducing infectious diseases such as malaria, which is particularly worse in rainy seasons in countries such as Malawi and Nigeria,^2^ kidney disease, liver disease, and cancer, etc.). Hence, blood donation shortages caused by the COVID-19 pandemic are wrecking Africa’s already overwhelmed blood transfusion services and are a guaranteed threat to a positive patient outcome, particularly for children under the age of 5, who are the recipients of 54% of the 118.5 million blood collected in low-income countries.^3^

In countries that rely on voluntary blood donations, particularly from students, the trends of blood supply and donation are likely to decline in many other countries if the COVID-19 pandemic continues. This will consequently and adversely affect beneficiaries in various hospitals, particularly in countries whose sole blood donors are volunteers and students.^4^

## What are the causes of reduced blood supply and donations?

Blood transfusions save lives, hence the need to maintain an adequate supply of blood. Blood donors have made it possible for blood to be available for transfusion to other patients. These donors can either be family members, voluntary non-renumerated donors, or paid donors. The World Health Organization preferentially recommends voluntary non-renumerated donors as the major source of blood for transfusion purposes.^5,6^ The uncertainty in the pattern of blood demand and supply and the inadequacy of human resources (which can be reduced due to ill health) are some of the most pressing challenges posed by the COVID-19 pandemic.^7^ The major contributors to low donations in Africa are restricted movements, lockdowns, and closure of blood donation institutions.^8^ Others include fear of contracting COVID-19 by both donors and healthcare workers due to limited knowledge on severe acute respiratory syndrome coronavirus 2 (SARS-CoV-2) transmission. In Zambia, for instance, there was a sharp decline in the number of blood donations because donors were afraid that by donating their blood, they could contract the virus. The lockdown imposed in the country was also a precipitating factor.^5^ However, the challenge might be more severe in countries where blood donation is not a culture. For instance, in Kenya, less than 1% of the 47 million Kenyans donated blood in the 2018/2019 year.^9^ In South Africa, there was a decreased blood supply and increased blood demand when schools and colleges were shut down because over 30% of the below 1% daily blood donors are younger than 25 years old.^10^

## What is the impact of decreased blood donations?

COVID-19 has caused some blood transfusion centres to miss their blood collection targets. For instance, the Zambia National Blood Transfusion Services target for the first quarter of 2020 was 18 750 units, but the institution barely collected 6516 units, representing 34.7% of the initial target.^5^ The Botswana Blood Transfusion Service had enjoyed an annual collection of 25 000 units, but due to COVID-19 preventive measures, only 11 000 units had been collected as of May 2020. This implied an impending decrease in the units of blood obtainable in a pandemic-affected year.^8^ The National Blood Transfusion Services in Botswana was able to collect only about 23 000 units of blood and blood products against the 45 000 units set as the target for the year 2020.^11^ In Kenya, COVID-19 has also reduced blood collection manpower (staff) due to ill health, social distancing measures placed in all institutions, and the lack of sufficient effective protective equipment to ensure staff and client safety.^9^

A study conducted at a teaching hospital in Nigeria revealed that 71.4% of patients with various types of malignancies such as acute myeloid leukaemia, bladder cancer, extra-orbital malignancy, prostate cancer, and lung cancer died due to the lockdown imposed in the country which disrupted the availability of blood donors.^12^ To this end, transfusion institutions – both hospital and independent blood transfusion units – have a key role to play in monitoring the demand and supply of blood and blood products to ensure that these products are adequately available.

## How do we address blood shortages?

When considering issues related to the availability of blood and blood products for clinical management, the principle of demand and supply should be evaluated.^13^

### Decrease demand

Proactive measures aimed at reducing demand and increasing the supply of blood and blood products must be implemented, particularly, at this time of the COVID-19 pandemic. These include patient blood management and strengthened cross-matching protocols for allogenic blood transfusion to reduce repeat transfusion.^10^

### Increase supply

To maintain supply, intense mobilisation campaigns and safer donation protocols are required. Mobilisation campaigns should be aimed at creating awareness: educating the populace on the importance of blood donations, debunking COVID-19-related blood donation myths, and sharing extra safety and screening measures instituted in the facilities.^14^

Unlike the recorded decline in the number of donated blood and blood products in Africa, India for instance devised ways to boost the confidence level of donors for outdoor blood donation drives during the lockdown period. This allowed the blood bank to receive adequate supplies of blood amid the pandemic.^14^ Some of the measures taken to boost the confidence of donors during the pandemic included the education of both staff and blood donors on COVID-19 preventive measures, the observation of safety protocols while conveying the donors to the venue of the blood donation drive, the provision of face masks and other personal protective equipment to all donors and staff, the compulsory use of hand sanitisers, and the use of infrared thermometers to check the body temperature of all the donors. Also, the use of ‘namaste’ as the mode of greetings to avoid handshakes was reinforced.^14^

Like other coronaviruses, SARS-CoV-2 infects the upper and lower respiratory tract and its RNA is shed into the serum or plasma of infected persons. Thus, some studies reported that SARS-CoV-2 can be detected in either blood plasma or serum.^15,16^ Theoretically, this implies that blood recipients can be infected with SARS-CoV-2 if they receive infected blood or blood products. However, Wang and his co-authors in 2020^15^ disagreed with this theoretical claim by saying: ‘to date, there are no reported cases of SARS-CoV-2 transmission by any blood product but transfusion transmission cannot yet be completely excluded’. Also, the World Health Organization has stated that the role of blood-borne transmission of SARS-CoV-2 is uncertain as the very low viral titres in plasma suggest a low risk of blood-borne transmission of the virus.^17^ Fortunately, there has not been any reported case of SARS-CoV-2 transmission via transfusion in any country.^15^ Consequently, it is not mandated for blood donors to get tested for SARS-CoV-2.^17^ However, because of the high number of asymptomatic COVID-19 infections, the safety of blood donors and staff needs to be ensured. Aside from the routine screening for infectious diseases, the screening of potential donors for COVID-19 exposure and symptoms should be done before donation, that is, at the time of donor clearance.

The World Health Organization recommends that donor restrictions be modified according to the COVID-19 community transmission status so as not to worsen the unavailability of donated blood. Some of the guidelines include sensitising donors about self-deferring upon the onset of symptoms, and instructing donors to inform the blood collection centre if they develop a respiratory illness. Deferring lasts for 14 days once a positive test is confirmed and delaying lasts for another 14 days after recovering from COVID-19 symptoms.^18,19^

### Improve management

Finally, blood inventory management should be implemented to prevent stockouts in emergency cases, collected blood and blood products should be harnessed judiciously.^14^

### Conclusion

COVID-19 is a threat to blood transfusion services in many countries, particularly lower-income countries. With a recorded decline in blood collection rates and missed targets, the impact casts a shadow on health and health outcomes amid the COVID-19 pandemic. COVID-19 offers African governments a chance to intensify efforts to effectively tackle all the uncertainties posed by the pandemic,^20^ implement responsive systems, and strategise effective ways to reach yearly targets even in pandemics.^21^ With steps taken to judiciously utilise available blood and broaden the sources of blood donation amid the pandemic, the impact of COVID-19 will be mitigated.
